# Epidemiological Trends of Dengue Disease in Mexico (2000–2011): A Systematic Literature Search and Analysis

**DOI:** 10.1371/journal.pntd.0003158

**Published:** 2014-11-06

**Authors:** Héctor Gómez Dantés, José Arturo Farfán-Ale, Elsa Sarti

**Affiliations:** 1 Instituto Nacional de Salud Pública, Cuernavaca, Morelos, México; 2 Hideyo Noguchi Institute, Mérida, Yucatán, México; 3 Sanofi Pasteur, Coyoacan, Mexico D.F., Mexico; University of Heidelberg, Germany

## Abstract

This systematic literature review describes the epidemiology of dengue disease in Mexico (2000–2011). The annual number of uncomplicated dengue cases reported increased from 1,714 in 2000 to 15,424 in 2011 (incidence rates of 1.72 and 14.12 per 100,000 population, respectively). Peaks were observed in 2002, 2007, and 2009. Coastal states were most affected by dengue disease. The age distribution pattern showed an increasing number of cases during childhood, a peak at 10–20 years, and a gradual decline during adulthood. All four dengue virus serotypes were detected. Although national surveillance is in place, there are knowledge gaps relating to asymptomatic cases, primary/secondary infections, and seroprevalence rates of infection in all age strata. Under-reporting of the clinical spectrum of the disease is also problematic. Dengue disease remains a serious public health problem in Mexico.

## Author Summary

Dengue disease is a tropical and subtropical mosquito-borne viral illness, and is a major public health concern in all endemic countries. Our aim was to determine the impact of dengue disease on the Mexican population over time, and to identify future research priorities and challenges of the surveillance system. To do this, we conducted a systematic literature review to describe the knowledge and gaps in the epidemiology of dengue disease. We used well-defined methods to search and identify relevant epidemiologic research conducted in Mexico between 2000 and 2011. This long-term review highlights an increase in the incidence of dengue disease—as well as in the number of severe cases and deaths in Mexico. Gaps in epidemiological knowledge regarding local serotype distribution, genotype evolution, age-stratified incidence and prevalence, hospitalization rates, underreporting rates as well as primary and secondary infections of dengue provide several avenues for future research. Improved epidemiological data from enhanced surveillance strategies (such as incorporating sentinel sites, more private health units, and information technologies) are required to enable evaluation of disease prevention and management interventions.

## Introduction

Dengue disease is the most prevalent arthropod-borne viral disease in humans [1]. It is caused by four serotypes of single-strand RNA flavivirus (dengue virus [DENV]-1, -2, -3, and -4), which are transmitted by blood-feeding mosquitoes – mainly *Aedes aegypti* (Linnaeus) [1].

The need to improve our understanding of the epidemiology of dengue disease is related to the challenges of ensuring adequate surveillance and effective control programmes, as well as to optimizing the anticipated future introduction of a vaccine. There is a large and increasing burden of dengue disease in tropical and subtropical regions of the world with a 30-fold rise over the past 50 years: an estimated 2·5 billion people are now considered to be at risk of the disease [2]–[4]. Approximately 50 million cases of dengue disease are estimated to occur annually, resulting in approximately 22,000 deaths [2]. A recent disease distribution model has estimated there to be 390 million dengue infections per year, of which 96 million manifest apparently (any level of clinical or subclinical severity) [3]. Understanding the transmission dynamics of dengue disease may also help to improve disease control programmes. Major increases in the incidence of dengue disease may be anticipated due to continued urbanization, the growing establishment of unplanned settlements in the urban areas of developing countries, and global warming [5]. Human movement has been identified as a key factor in determining the transmission dynamics of dengue disease [6]–[8], and urbanisation without adequate water management has encouraged its spread [9].

Mexico contributes to the high number of dengue cases in the Americas [10] and provides favourable conditions for the spread of dengue disease. The country is heavily populated and its population has increased rapidly (from 97 million in 2000 to >112 million in 2010 [11]) and it has large tropical and subtropical regions. In addition, it has high levels of foreign trade and tourism, which encourage human movement further increased by intensive migration from central American countries [12]. Rapid urbanisation during the past 20–30 years has also encouraged the spread of dengue disease throughout Mexico [13].

Control strategies instigated in the 1950s and 1960s resulted in the near eradication of *Ae. aegypti* in the Americas. In 1963, *Ae. aegypti* was reported as having been eradicated from Mexico and, although the mosquito reappeared only 2 years later, the country was free from dengue disease from the early 1960s until 1978 [14]. Re-emergence and dissemination of dengue disease went practically unnoticed until major epidemics hit the east coast of Mexico during 1979 and 1980, caused by the DENV-1 serotype [15]. During the 1980s, there was a general decline in the identification of dengue disease cases in Mexico relative to the epidemics of 1979 and 1980, but in the mid-1990s a resurgence of cases coincided with the emergence of the DENV-3 serotype [16]. Recent control strategies have had little impact, as shown by the lack of reduction in the incidence of dengue disease [17]. Traditional vector control strategies have several limitations in terms of economic and resource costs, coverage and delivery and sustainability [18]. It is essential that maximum benefit is gained from the available resources, and epidemiological understanding is crucial for determining the populations, areas, and times of greatest risk for dengue disease.

The National Epidemiological Surveillance System (SINAVE), is a nationwide regulatory body that observes, facilitates, promotes and guides epidemiological activities in the country and embodies a series of standardized methods and processes aimed at timely and uniform epidemiologic surveillance information provided by several health institutions from the public and private sectors and is regulated and coordinated by the Ministry of Health. The SINAVE comprises a system that collects uniform data about population health, risks, and the assessments healthcare plans and programs at all levels (national, state, jurisdictional, and local): The Unified Epidemiological Surveillance Information System (SUIVE), has a series of tools to expedite data collection, analysis, interpretation and dissemination processes regarding specific health problems reported from almost 20,000 health units disperse in the country supported by a national diagnostic laboratory network and alternative data sources.

The surveillance of the Vector Borne Diseases includes detection, notification, epidemiological and clinical study as well as follow-up of the cases and every death of each new case detected by the physician in any medical health unit.

Dengue surveillance is mandatory and severe dengue demands immediate notification (<24 hrs. of diagnosis). Dengue clinical case definitions include suspected or probable cases while the confirmed category demands laboratory diagnosis either by serology or virus isolation. The national surveillance system in Mexico comprises obligatory weekly reporting of suspected and probable dengue disease cases, using an established surveillance protocol. This system has evolved from a clinically based reporting system that was developed in the 1990s; it is now more robust, with support from a national network of diagnostic laboratories. Since 2005, only laboratory-confirmed cases have been reported in the Mexican and Pan-American Health Organization (PAHO) official bulletins and associated web pages. In 2007, a change in the reporting system made laboratory confirmation mandatory for all probable dengue disease cases during periods of low transmission and 30% of probable cases in an outbreak situation (the proportion of samples confirmed to be positive is applied to the remaining probable cases and reported as the estimated number of cases). All severe cases and deaths need to be confirmed by serology or virus isolation [19].

Information reported to PAHO or WHO only includes confirmed cases but for surveillance purposes the Ministry of Health uses probable as well as confirmed cases to analyze local, state and national dengue patterns and trends. The evaluation, monitoring and assessment of the overall surveillance process is performed on a monthly basis following the report of several indicators that describe opportunity and coverage of reports, integrity of epidemiological, clinical and laboratory data, quality of blood samples and laboratory diagnosis data. Since the information provided by the national surveillance system is the most important data source for the analysis of dengue epidemiology in the country we included the period (2000–2011) with the most consistent and systematic data since 2000.

This article describes the more recent epidemiology of dengue disease in Mexico between 2000 and 2011, and aims to identify gaps in epidemiological knowledge and future research needs.

## Materials and Methods

A Literature Review Group, which included external experts in dengue disease as well as the sponsor, developed a protocol for this literature survey and analysis based on the preferred reporting items of systematic reviews and meta-analyses (PRISMA) guidelines and guided the search and selection process described below. The protocol was registered on PROSPERO, an international database of prospectively registered systematic reviews in health and social care managed by the Centre for Reviews and Dissemination, University of York (CRD4201200217: http://www.crd.york.ac.uk/PROSPERO/display_record.asp?ID=CRD42012002127) on 9 March 2012.

We conducted a systematic literature review to describe the available epidemiology of dengue reported in Mexico between 1 January 2000 and 23 February 2012. Our objectives were to describe the recent epidemiology of dengue (national and regional incidence [by age and sex], seroprevalence and serotype distribution and other relevant epidemiological data) and to identify gaps in epidemiological knowledge requiring further research and challenges of the surveillance system. We chose to begin our review on 1 January 2000 as opposed to an earlier date, as we hypothesised that one decade of data would provide an accurate image of the evolution and periodicity of dengue outbreaks and limit any bias due to changes in surveillance practices mentioned early. This period would also enable the observation of serotype distribution over time and through several epidemics. We also chose to include surveillance data reports to supplement the limited data available from published sources in order to better determine the burden of disease, the clinical spectrum and its potential impact on transmission.

We utilised an inclusive search strategy to find papers, theses, dissertations, reports and statistical tables, as well as to official web sites and grey materials. It was expected that the resulting articles would be heterogeneous with respect to data selection, and classification of cases, and would not be methodologically comparable. We therefore planned not to perform a meta-analysis.

### Search strategy and selection criteria

The databases searched included Medline, PreMedline, Excerpta Medica Database (EMBASE), Scientific Electronic Library Online (SciELO), Virtual Health Library (VHL), the WHO Library database (WHOLIS), Mexican university databases, and Mexican hospital/public health databases. Relevant reports and guidelines were gathered from online sources such as the Mexican Ministry of Health surveillance reports. Mexican periodicals were searched and general Internet searches (e.g., Google) were performed to identify relevant ‘grey’ literature (e.g., lay publications). Search strings for each database were designed with reference to the expanded Medical Subject Headings thesaurus, encompassing the terms ‘dengue’, ‘epidemiology’, and ‘Mexico’. Different search string combinations were used for each electronic database with the aim of increasing the query's sensitivity and specificity. All searches were conducted between 9 and 23 February 2012.

The inclusion/exclusion criteria were defined by the Literature Review Group. Decisions relating to inclusion/exclusion disputes were made by reaching a consensus via teleconferences. Only studies published in English and Spanish between 1 January 2000 and 23 February 2012 were included, providing they included epidemiological data relating to age, sex and serotype distribution, or the seroepidemiology or seasonality of the disease in Mexico. For databases that did not allow language and/or date limitations, references not meeting these criteria were deleted manually at the first review stage. No limits by sex, age and ethnicity of study participants or by study type were imposed, although single-case reports were excluded, as were studies that only reported data for the period before 1 January 2000 as were publications of duplicate data sets (e.g., in meta-analyses and other reviews), unless the articles were reporting different outcome measures. Additional publications not identified by the search strategy, and unpublished data sources meeting the search inclusion criteria were included if recommended by a consensus of the Literature Review Group.

After removing duplicate citations, the Literature Review Group reviewed the titles and abstracts and identified those for which the full text was retrieved. The final selection of relevant articles was made by the Literature Review Group following a second review of the full text to ensure compliance with the search inclusion and exclusion criteria. We did not formally rank articles and other data sources nor exclude them based on the quality of evidence. We recognize that an assessment of study quality can add value to a literature review, however, given the expected high proportion of surveillance data, the consensus among the Literature Review Group was that, given available data sources and the nature of surveillance data (passive reporting of clinically-suspected dengue), such quality assessment would not add value in this case or could even restrict the information to very few articles and documents.

The selected data sources were collected and summarized using a data extraction instrument developed as a series of Excel (Microsoft Corp., Redmond, WA) spreadsheets. Data for analysis were extracted into the spreadsheets according to the following categories: incidence, age, sex and serotype distribution, serotype data, seroepidemiology or seasonality and environmental factors, by national or regional groups. Data from literature reviews of previously published peer-reviewed studies and pre-2000 data published within the search period were not extracted. Following data extraction and checking, all members of the Literature Review Group were provided with all original data sources and the extraction tables for review and analysis.

## Results

**Figure 1:**
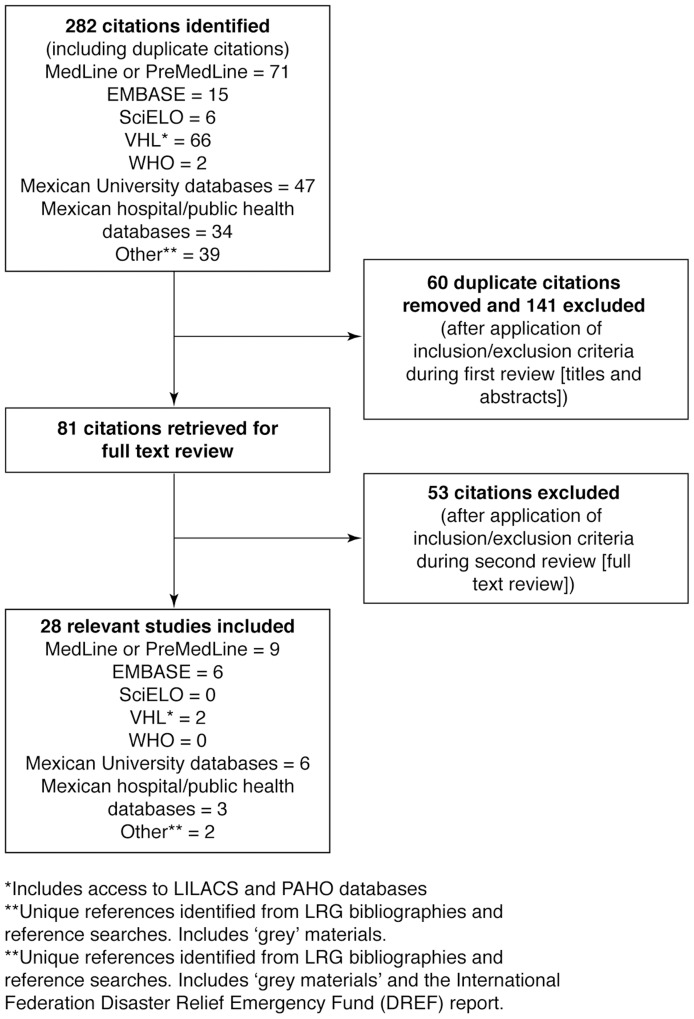
Evaluation of studies according to the preferred reporting items of systematic reviews and meta-analyses (PRISMA). All references identified in the online database searches were assigned a unique identification number. Following the removal of duplicates and articles that did not satisfy the inclusion criteria from review of the titles and abstracts, the full papers of the first selection of references were retrieved either electronically or in paper form. A further selection was made based on review of the full text of the articles.

The search identified 282 data sources, of which 28 fulfilled the inclusion criteria (Figure 1; Table S1). Of these, 20 were articles in journals although none of these were population studies that provided national prevalence data. The remaining data sources were either surveillance reports that were found during the initial searches or statistical tables recommended or accessed by members of the Literature Review Group to supplement the incomplete national data. The lack of population studies reflects the scarce sources of information and the dependence on an incomplete surveillance system in Mexico: epidemiological data sources are centralized by the Ministry of Health; only confirmed cases are reported and data from public health laboratories are integrated into the information system. A narrative synthesis of our findings is presented.

### Disease burden: What do we know and where are the evidence gaps?

#### National epidemiology

**Figure 2:**
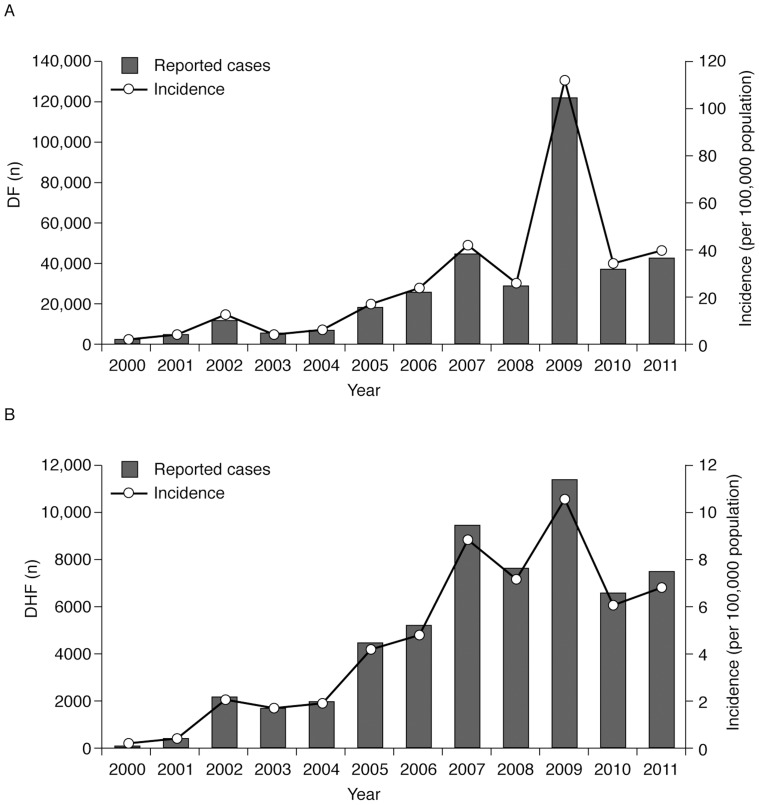
Reported number and incidence of dengue fever and dengue haemorrhagic fever cases, Mexico, 2000–2011 [20]. Over the period of the literature survey (2000–2011), the overall annual number of confirmed cases of (A) dengue fever (DF) and (B) dengue haemorrhagic fever (DHF) increased considerably in Mexico. The increase in number of cases and population incidence over time was not linear and peaks were observed in 2002, 2007, and 2009.

Because of the lack of population studies that provided national prevalence data we are reliant on Mexican public health data to provide an assessment of the national epidemiology of dengue and to identify trends over time. Consequently, despite the risk of introducing selection bias it is useful to examine the public health data made available during the review period. Over the period of the literature survey (2000–2011), the overall annual number of confirmed cases of dengue disease and severe dengue disease increased considerably in Mexico. According to the public health data, between 2000 and 2011 there were approximately 316,000 dengue fever (DF) cases nationally. The annual number of uncomplicated cases was 1714 in 2000 and 15,424 in 2011. However, the increase over time in the annual number of cases was not smooth and peaks were observed in 2002, 2007, and 2009 (Figure 2A) with a large increase in cases over the period 2004–2007. The population incidence of DF increased from 1·72 per 100,000 population in 2000 to 14·12 in 2011, with a peak of 112 in 2009 (Table 1; Figure 2A) [20].

**Table 1:** Incidence of dengue disease (per 100,000 population) in Mexico and associated hospitalization and mortality: national data [15], [16], [20]–[25].

	Year											
	2000	2001	2002	2003	2004	2005	2006	2007	2008	2009	2010	2011
DF incidence [20] *	1·72	4·6	12·9	5	5·93	16·43	22·94	40·59	26·26	112·18	33·9	14·12
Aldana Cruz 2011 [21]		5	12·5	5	6·5	17	22	40·5	23			
García Vega 2009 [22]									108·5			
DF-related hospitalization (no. of cases) [20] *	172†	236†	2181†	1591†	1992	6026	5521	10,962	6935	9627	5528	6906
DHF incidence [20] *	0·07	0·31	2·1	1·7	1·86	4·15	4·81	8·92	7·11	10·6	6	5·88
DF/DHF ratio (no. of cases) [20] *	25·6	14·9	6·13	2·94	3·18	3·95	4·76	4·55	3·69	10·64	5·62	2·40
Cisneros Solano 2005 [24]	500	32·3	5·18									
Falcón-Lezama 2009 [16]		20	7·41	3·66	8·33	4·52	5	5·13				
Diaz 2006 [15]						4·29						
Torres 2007 [25]						3·97						
International Federation Disaster Relief Emergency Fund 2009 [23]										5·29		
DHF-related hospitalization: no. of cases [20] *	44†	168†	2532†	1188†	1837	4175	3488	7543	4699	9658	4740	1817
DHF-related mortality: no. of deaths (lethality = deaths over total no. of DHF) [20] *	0 (0%)	1 (0·32%)	7 (0·32%)	11 (0·62%)	13 (0·67%)	41 (0·93%)	20 (0·39%)	29 (0·31%)	36 (0·47%)	93 (0·82%)	76‡ (1·16%)	50 (0·99%)
Cisneros Solano 2005 [24]			6 (0·4%)									

*Mexican public health data [20].

† Social security sector excluded.

‡ 10 additional deaths classified as dengue fever (A90X) occurred in 2010.

There is considerable variation in the reported incidence of dengue disease across the different publications retrieved (Table 1) [15], [16], [20]–[25]. For example, reports of the population incidence of DF in 2008 ranged from 23 to 108·5 per 100,000 population [20]–[22]. Data provided by the Disaster Relief Emergency Fund (DREF) [23] indicated lower numbers of DF and dengue haemorrhagic fever (DHF) cases in 2009 (41,687 and 7898, respectively) than Mexican public health data (120,649 and 11,396 cases) [20]. Vázquez-Pichardo et al. [26] reported a much higher number of DF cases for 2010 (127,840) than the public health data (36,740 cases).

As with uncomplicated DF, a major increase in the annual number of cases of DHF was apparent, with ≤100 in 2000 to almost 6500 in 2011 (Figure 2B). The annual ratio of uncomplicated DF per case of DHF varied considerably between 2000 and 2011: it was highest in 2000 (25·6) and then decreased to levels of about 5 for most of the period, except in 2009 when the ratio peaked at 10·6 (Table 1). The proportion of severe cases reported from the overall number of dengue disease cases increased from <5% in 2000 to 41% in 2011 [20]. However, this does not necessarily mean that severity of dengue disease has increased in Mexico, as the surveillance system may have become more sensitive for the detection (notification) of severe cases. Most surveillance systems detect a higher proportion of severe cases of dengue disease than less prominent uncomplicated cases.

Hospitalizations for the period 2004–2010 attributable to uncomplicated DF followed a slightly different pattern from DF incidence (Table 1). The number of DF hospitalizations in 2005 (6026) was slightly higher than the number in 2010 (5528), indicating no increase in severity of DF cases in 2010 *vs* 2005, whereas there were twice as many DF cases in 2010 *vs* 2005 (36,740 *vs* 17,487) [20]. The lethality rate for DHF was kept under international standards (<1%) over most of the survey period, ranging from zero in 2000 to 0·99% in 2011 [20].

#### Regional epidemiology

**Figure 3:**
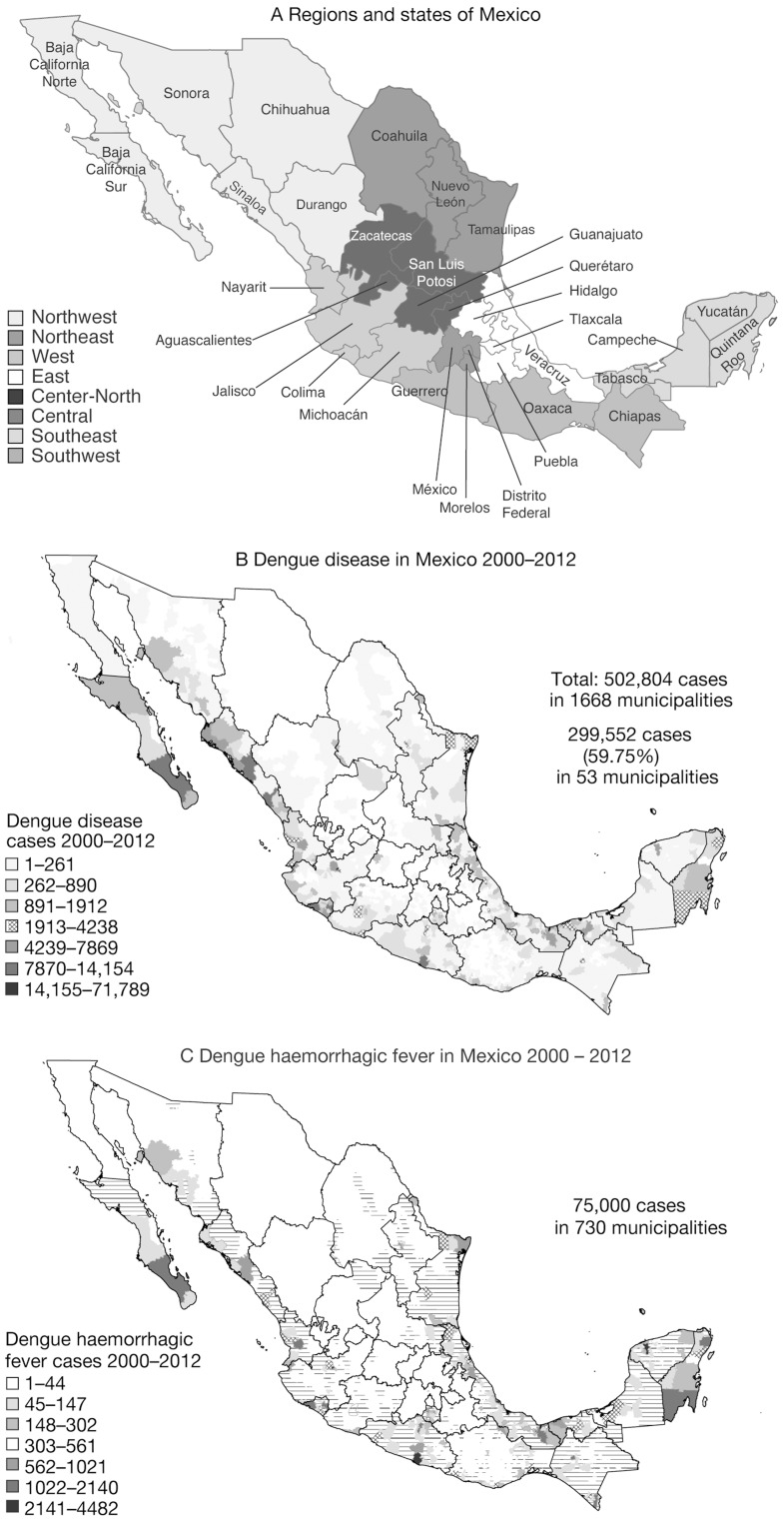
Geographical distribution of dengue disease and dengue haemorrhagic fever cases in Mexico, 2000–2012 [19], [20]. Regional populations in Mexico are exposed to differing magnitudes of dengue disease risk due to the mix of epidemic, endemic, and hyperendemic areas. Mexico is divided into 31 states and one federal district that contains the capital, Mexico City (A). These dispersion maps of dengue disease and DHF throughout Mexico highlights the regional pattern of the occurrence of (B) dengue disease and (C) dengue haemorrhagic fever with high concentrations of cases in the important tourist and commercial centres on the coast and in tropical areas.

The epidemiological spectrum of dengue disease in Mexico is a mix of epidemic, endemic, and hyperendemic areas, with regional population areas exposed to differing magnitudes of risk. Differences in case definitions, reporting criteria (confirmed and/or clinical cases), or laboratory diagnostic support may cause variations in the reported incidence of dengue disease, either between locations or over time. Nevertheless, detailed analysis (incorporating the most recent 2012 data) demonstrates the dispersion of dengue disease and DHF throughout Mexico and highlights the very clear regional pattern of the incidence of dengue disease (Figure 3), with high concentrations of cases in the coastal and tropical areas (50% of dengue disease cases were concentrated in only 65 municipalities or counties). Coastal areas are important tourist and commercial centres and the areas of highest DF incidence were on the Gulf of Mexico coast (2006) and on the northern Pacific coast (2008 and 2010), with high incidence observed on the Yucatan peninsula (Southeast Region) in 2006, 2010 and 2011. Veracruz in the Gulf Coast Region had the largest number of DF cases (Table 2) and highest numbers of DHF cases (data not shown) [20]. The national tendency towards an increase in the number of DF cases over time, with larger peaks in 2007 and 2009, was less clear at the state level, as regional peaks in case numbers mask the overall national pattern.

**Table 2:** Dengue disease in Mexico: regional data from public health databases [20].

State [Region]	2000	2001	2002	2003	2004	2005	2006	2007	2008	2009	2010	2011
**Northwest Region**												
Baja California	1 (0·0)										9 (0·3)	20 (0·6)
Baja California Sur	1 (0·2)	1 (0·2)	9 (2·2)	486 (101·9)	2 (0·4)	10 (2·0)	88 (17·1)	33 (6·1)	209 (37·9)	519 (91·8)	1946 (335·9)	63 (10.6)
Sonora	328 (14·5)	0 (0)	259 (11·2)	1179 (48·9)	25 (1·0)	72 (2·9)	86 (3·4)	14 (0·6)	1083 (43·5)	456 (18·2)	4130 (163·1)	87 (3.4)
Sinaloa	9 (0·4)	185 (7·4)	566 (22·4)	1241 (45·6)	122 (4·4)	653 (23·6)	416 (14·9)	546 (20·6)	1120 (42·3)	2439 (91·9)	1091 (41·1)	91 (3.4)
**North Region**												
Chihuahua								1 (0·0)	3 (0·1)		2 (0·1)	
Durango	1 (0·1)						122 (7·8)	329 (21·4)	65 (4·2)	297 (19·2)	18 (1·2)	12 (0.8)
Aguascalientes											2 (0·2)	3 (0·26)
Coahuila		1 (0·0)	236 (9·7)	4 (0·2)	3 (0·1)	120 (4·7)	228 (8·9)	813 (31·6)	29 (1·1)	538 (20·5)	85 (3·2)	5 (0·2)
Nuevo Leon	22 (0·6)	14 (0·4)	18 (0·4)	36 (0·9)	36 (0·9)	517 (12·2)	133 (3·1)	2734 (63·0)	803 (18·3)	612 (13·8)	2255 (50·1)	715 (15·7)
Zacatecas								156 (11·3)	2 (0·1)			0
San Luis Potosí			3 (0·1)		2 (0·1)	169 (7·0)	273 (11·3)	243 (9·9)	265 (10·7)	3322 (133·7)	315 (12·6)	81 (3·2)
**West Region**												
Colima	2 (0·37)	2 (0·4)	1766 (310·7)	30 (5·2)	6 (1·0)	362 (61·2)	1390 (232·2)	777 (132·7)	2139 (360·6)	6479 (1078·2)	1151 (189·1)	373 (60·5)
Nayarit	2 (0·21)		1015 (103·6)	90 (9·14)	153 (15·4)	408 (40·9)	537 (53·5)	1033 (107·1)	305 (31·54)	10,064 (1038·0)	1642 (168·9)	54 (5.5)
Jalisco			1270 (19·0)	33 (0·5)	30 (0·4)	88 (1·3)	1605 (23·4)	869 (12·6)	1430 (20·5)	55,289 (788·0)	1730 (24·5)	219 (3·0)
Michoacan	12 (0·3)	41 (0·9)	381 (8·7)	107 (2·5)	90 (2·1)	37 (0·9)	416 (9·8)	2297 (57·5)	2566 (64·5)	4740 (119·6)	1715 (43·4)	577 (14·7)
**Central Region**												
Hidalgo			32 (1·3)	3 (0·1)	120 (5·1)	687 (29·0)	212 (8·8)	44 (1·8)	579 (24·0)	1319 (54·5)	45 (1·8)	50 (2·1)
Puebla	51 (1·0)	35 (0·7)	13 (0·2)		27 (0·5)	50 (0·9)	228 (4·1)	307 (5·54)	601 (10·7)	595 (10·5)	1227 (21·5)	174 (3·0)
Tlaxcala												0
Guanajuato								636 (12·7)	14 (0·3)	197 (3·9)		
Queretaro							110 (6·7)	8 (0·5)	3 (0·2)	36 (2·1)	1 (0·1)	76 (4·3)
Distrito Federal*	1* (0·0)											0
Mexico		37 (0·3)	61 (0·4)		29 (0·2)		7 (0·0)	44 (0·3)	58 (0·4)	261 (1·8)	41 (0·3)	6 (0·04)
Morelos		14 (0·9)	102 (6·2)	77 (4·6)	57 (3·4)	229 (13·3)	2425 (139·7)	1119 (67·9)	5943 (357·6)	1073 (64·1)	1486 (88·1)	767 (45·1)
**Gulf Coast Region**												
Tamaulipas	221 (8·1)	47 (1·7)	147 (5·2)	9 (0·3)	99 (3·2)	5230 (165·3)	152 (4·7)	1412 (45·3)	1014 (32·1)	1113 (34·9)	826 (25·6)	85 (2·6)
Veracruz	570 (8·0)	2244 (31·3)	2357 (32·7)	1084 (14·9)	4250 (58·4)	3901 (53·5)	6991 (95·6)	12,608 (174·1)	2069 (28·5)	9905 (136·1)	1004 (13·8)	1257 (17·2)
Tabasco	3 (0·1)	88 (4·5)	327 (16·3)	100 (4·9)	73 (3·6)	368 (17·8)	174 (8·3)	1695 (83·5)	870 (42·6)	5810 (283·3)	488 (23·7)	321 (15·5)
**Southeast Region (Gulf and Caribbean)**												
Campeche	3 (0·4)	53 (7·3)	372 (50·5)	17 (2·3)	26 (3·4)	93 (12·0)	83 (10·5)	217 (27·9)	46 (5·8)	766 (96·2)	1164 (144·6)	935 (114·8)
Yucatan		252 (14·8)	749 (43·4)	19 (1·1)	51 (2·9)	123 (6·8)	465 (25·4)	1474 (78·6)	573 (30·2)	3528 (183·5)	2198 (112·9)	5366 (272·4)
Quintana Roo	17 (2·1)	291 (34·6)	486 (56·2)	102 (10·0)	239 (22·7)	665 (60·9)	1781 (157·6)	3545 (290·4)	421 (33·2)	790 (60·1)	2053 (150·7)	1762 (124·9)
**South Pacific Region**												
Guerrero	14 (0·4)	521 (16·2)	2191 (67·1)	391 (12·1)	270 (8·3)	1012 (31·04)	3336 (102·0)	3292 (104·5)	3311 (105·3)	5229 (166·5)	5737 (183·0)	710 (22·7)
Oaxaca	204 (5·7)	446 (12·3)	570 (15·6)	171 (4·6)	147 (3·9)	1019 (27·4)	2866 (76·7)	4465 (125·6)	1341 (37·7)	2191 (61·7)	3529 (99·4)	950 (26·8)
Chiapas	252 (6·2)	371 (9·0)	324 (7·7)	40 (0·9)	386 (8·8)	1674 (37·9)	537 (12·0)	2225 (50·4)	1153 (25·8)	3081 (68·4)	850 (18·7)	665 (14·5)

Data shown are number of uncomplicated dengue fever cases (incidence per 100,000 population).

*Cases in Distrito Federal are imported from other Mexican states (no dengue disease transmission occurs).

The highest numbers of hospitalizations due to DF and DHF were in Veracruz during 2004, 2007, and 2008, Guerrero in 2006 and 2010, Jalisco in 2009, and Quintana Roo and Yucatán in 2011. States with the highest numbers of dengue disease-related deaths were Veracruz (2007; n = 10), Morelos (2008; n = 16), Jalisco (2009; n = 48), Guerrero (2010; n = 16) and Yucatán (2011; n = 25). As with the distribution of DF and DHF cases, the highest numbers of dengue disease-related hospitalizations and deaths tended to occur in the coastal states [20].

Three individual studies retrieved by the literature survey reported state-specific numbers of DF cases over sequential years [27]–[29]. Although the numbers reported in these studies differed from the public health data the temporal patterns were similar. Two different epidemiological patterns of transmission have been observed: an endemic pattern in Gulf of Mexico states, and a seasonal pattern on the Pacific coast and Yucatan peninsula [15], [30]. Before 1987, the highest altitude at which *Ae. aegypti* had been recorded in Mexico was 1630 m above sea level, and it may now be encountered at altitudes of up to 2130 m. Increased temperatures relating to climate change could explain the increase in the altitude ceiling [12], [31].

#### DENV serotype distribution

**Figure 4:**
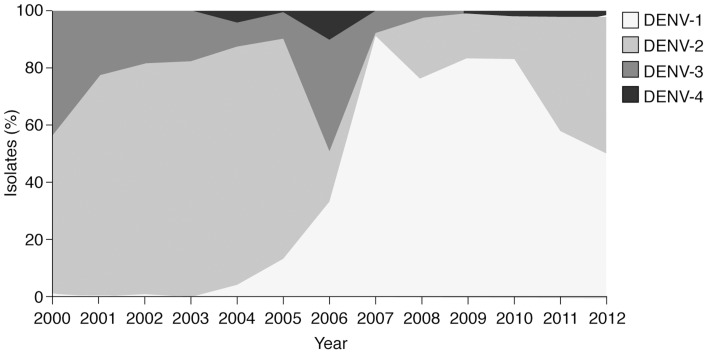
Circulation of dengue virus serotypes in Mexico, 2000–2011 [16], [20], [26]. Data shown are number cases (incidence per 100,000 population). All four DENV serotypes were in circulation during the review period, although the predominant serotype varied between years: at the beginning of the review period, DENV-2 was the predominant serotype (2000–2005). DENV-1 became predominant in 2006–2010, after which DENV-1 and -2 isolates were present in similar proportions. DENV-3 isolates were more common before 2007. DENV-4 isolates were present at low levels in most years after 2003.

All four DENV serotypes have been shown to circulate in Mexico at different times (Figure 4) [16], [20], [26], creating epidemic, endemic, and hyperendemic scenarios. According to a study by Falcón-Lezama et al., DENV-2 was the predominant serotype from 2000 until 2005, and DENV-1 became predominant in 2006 [16], although Rivera Osorio reported that DENV-3 was the most commonly isolated serotype in 2006 [32]. Vázquez-Pichardo et al. reported serotype data gathered by Instituto de Diagnóstico y Referencia Epidemiológicos (InDRE) following the introduction of a new diagnostic algorithm with improved dengue serotype identification in 2008 [26]. Data for 2009 and 2010 show the continued predominance of DENV-1 (83% in both of these years), with DENV-2 representing most of the remaining cases (17% in 2009 and 16% in 2010). The percentage of DENV-4 isolates was less than 1% in 2009 and 2010 [26]. For the period 1995–2003, Navarette-Espinosa et al. reported that 9% of isolates were DENV-1, 60% were DENV-2, and 31% were DENV-3 (there were no DENV-4 isolates) [33].

Exact relationships between infection with DENV serotypes and different forms of clinical disease are multifactorial and not fully understood. Fluctuations in serotype predominance may have important implications for the incidence and severity of dengue disease. For example, an increase in the incidence of both DF and DHF in 2006–2007 coincided with the emergence of DENV-1 as the predominant serotype [16]. The genotype of DENV serotypes may also play a role in the severity of dengue disease outbreaks. For example, the increase in DHF in 2001 has been linked to the introduction of the American–Asian genotype of DENV-2, and in a dengue disease outbreak in Yucatan State in 2002 that was attributable to this genotype as many as 31% of confirmed cases were classed as DHF [34].

Unfortunately, there is no systematic approach to identifying the predominance of dengue virus serotypes or genotypes in Mexico. The available data are based on positive isolates from areas where surveillance is most effective (e.g., where an outbreak increased the collection of samples from individuals with acute DF). Consequently, it is not possible to be certain of the nationwide predominance of serotypes, and no precise data on the geographical distribution of each serotype are available.

#### Seroprevalence

There have been no recent national sero-surveys to identify susceptible or infected populations, and no field studies have investigated the number of asymptomatic cases, either during an outbreak or in a low transmission situation. Consequently, neither the prevalence of infection in the overall population in Mexico nor the risk factors for severe disease are known, other than that previous exposure to a different serotype increases the risk of severe dengue disease [35].

Our search retrieved several regional, cross-sectional studies of DENV seroprevalence. Considerable variability was observed between studies: seroprevalence was reported to be low in Villahermosa (Tabasco state University) in 2005 (9·1%; age range 18–30 years) [36] and moderate in Colima (Colima) in 2001–2002 (32·8%; all ages) [37]; high seroprevalence was reported in Matamoros in 2004 and 2005 (78·3% [38] and 76·6% [39]; age ranges ≥5 and ≥15 years, respectively) and in Jaltipan (Veracruz) in 2003 (79·6%; all ages) [40]. Factors potentially contributing to the variability include modest sample sizes and differences between studies in participant selection. In addition, there is a tendency to observe greater dengue activity in epidemic than in non-epidemic areas since the more samples are collected for laboratory testing in areas with higher transmission. Slightly more females than males appear to have been infected with dengue virus in Mexico, and seroprevalence appears to increase rapidly with age during childhood [38]–[40]. Navarrete-Espinosa et al. reported seropositivity rates of 17% among those aged less than 1 year, 69% in those aged 5–14 years, 79% in those aged 15–24 years, and 86–94% among older age groups [40].

#### Age, sex, and seasonal distribution

The distribution of dengue disease by age group and sex is an important aspect of the clinical picture, particularly for identifying the population most at risk. The general pattern for age distribution is an increase in the number of DF cases during childhood, with the incidence peaking between the ages of 10 and 20 years. This is followed by a gradual decrease during adulthood, with individuals aged over 65 years usually having the lowest incidence (Table S2). Similar age-group distribution patterns were observed for DHF, and dengue-related deaths have been shown to occur among all age groups (with a tendency to peak among those aged 15–29 years) [20].

Several publications provided age-group distributions of dengue disease (cases of DF and DHF pooled) on a regional level, and some variation between different regions was apparent. In Oaxaca, more than 50% of all cases were reported among children and adolescents aged under 15 years [41]–[43], compared with 25–40% in Sinaloa [44], Yucatan [34], Colima [45], and Hidalgo [21]. In Yucatan [34], Colima [45], and Oaxaca [42], 11–13% of cases were reported among adults aged 45 years or older compared with 21% in Hidalgo [21] and 25% in Sinaloa [44]. More females than males appear to be affected by DF in Mexico, although the difference between the sexes decreased between 2003 and 2010 [20]. In 2003, there were almost 50% more DF cases among females, in 2010 this percentage was below 20%, and in 2011 the percentage was almost the same (53% females). More cases of DHF were reported among females than among males during 2003–2005, but there were more cases among males during 2006, 2009, and 2010 [20]. Assessing the validity and comparability of the data from the various sources used in this review has proved challenging. In particular, many data from individual studies (for example those relating to age and sex distribution) tend to reflect the relevant demands on the health service of different age groups or sexes. Improvements to the surveillance system are required to obtain a more complete picture of the epidemiology of dengue in Mexico.

A number of studies reported a seasonal pattern of dengue disease, with most cases occurring between September and December [22], [46]. Despite the seasonal pattern, significant numbers of dengue disease cases are now reported throughout the year [20]. For example, in 2010 monthly totals for uncomplicated DF cases ranged from 1484 to 2448 during January to April, with 4224 cases in November and 686 in December [20]. Historically, it was assumed that the arrival of the wet season (May to October), triggered the beginning of the *Ae. aegypti* breeding season, but the data from recent years suggest that *Ae. aegypti* has begun to reproduce all year round [12].

## Discussion

The present literature survey provides a valuable overview of the available dengue disease data for Mexico over the period 2000–2011. The information available on dengue disease in Mexico is improving but it is not yet sufficient, there is a need for improved epidemiological data to understand the dynamics of dengue disease transmission. As in many surveillance systems worldwide, only cases that present to the Mexican health service are identified as dengue disease. Consequently, many cases, which may be symptomatic, subclinical, or asymptomatic, may not be reported and so the true incidence of dengue infection is unknown. Whilst the level of symptomatic disease drives healthcare-seeking behaviour and is consequently a very relevant marker for public health systems, nevertheless an understanding of the overall level of infection across a variety of epidemiological settings (epidemic, endemic, and hyperendemic) is required to evaluate the current situation and the impact of control interventions (e.g., vector control, behavioural measures or, in the future, vaccination).

A number of differences were observed between Mexican public health reports and data retrieved from other sources, and it would be interesting to conduct a separate for the analysis between data of studies with defined method versus data gathered from national reporting. One reason for the observed differences may be due to a potential under-reporting of the number of cases of dengue disease by the surveillance system in Mexico. Surveillance in Mexico is designed to detect probable or suspected cases but only laboratory-confirmed cases have been reported in Mexico since 2005, which may explain the changes in reported cases since that year. Despite their critical role in the assessment and monitoring of disease burden, routine surveillance systems are not designed to detect all cases of dengue disease [47]. To estimate the real number of cases, reported cases need to be multiplied by an expansion factor (EF) [48], a ratio between projected and reported numbers of cases that represents the degree of underreporting. It has been proposed that reported data for hospitalized and fatal cases of dengue disease for Mexico should be multiplied by an EF of 2·3 to gain an improved indication of the actual number of cases [48], [49]. For cases managed in an ambulatory setting, this EF is estimated to be 15 [48], [49]. In a cohort study of dengue disease transmission, 155/254 (61·0%) recent DENV infections were asymptomatic, and only 18/99 (18·2%) of symptomatic infections were reported on the surveillance system [50]. Thus, the clinical spectrum of dengue disease in Mexico, and consequently the overall burden of disease, are probably not completely reflected by the national surveillance data. Reporting bias (i.e., under-reporting of uncomplicated DF) may also explain the low DF∶DHF ratios between 2002 and 2011, which are mostly about 5; this ratio is expected to be in the region of 7–8 [27]. The key issue is that as uncomplicated cases of DF may be asymptomatic they are liable to remain unreported, whereas DHF cases are always symptomatic. Another consideration is that hospitalization data for 2000–2003 do not include cases from the social security sector.

Possible contributors to the differences observed between Mexican public health data and the data from other sources also include differences between institutions (e.g., in terms of interpretation and reporting of source data), and time elapsing before the data for a period of time are reported. For example, the Mexican Ministry of Health initially receives data 2 weeks after the occurrence of dengue disease but data provided this early are liable to adjustment as suspected cases are confirmed; it usually takes several weeks for dengue disease data to be finalised. Where there are differences between different data sources, it is difficult to define the real burden of dengue disease. Adjustments in the methods of data collection and case definitions mean that patterns observed over time during the survey period (2000–2011) should be interpreted with caution. For example, in 2010, a system for classifying cases as estimated, confirmed and probable was introduced. Little information is available on asymptomatic DENV infection, rates of primary/secondary infection or dengue infection in laboratory data. An extensive, nationwide seroprevalence survey would clearly be valuable in this regard.

The geographic distribution of dengue disease reflects regional patterns in transmission, which may result from climate characteristics such as temperature, and humidity, geographical effects (e.g., the elevational distribution of *Ae. aegypti*) and environmental factors such as population density and human movement as a result of tourism, working migrants, and illegal migration from Central American countries. Diaz et al. discussed the geographical spread of dengue disease in Mexico [15]. They commented that the southern states are often affected first and with the highest incidence, attributing this to the introduction of dengue virus from countries to the south of Mexico. The same authors cited serotype circulation within the country as another reason for the persistence of dengue disease within Mexico. Climate change could also be contributing to the phenomenon of *Ae. aegypti* beginning reproduce all year round and in recent years natural disasters have increased in frequency, so that flooding has become more likely in August and September, facilitating vector reproduction during the following months.

As in other countries, serotype circulation within Mexico is undoubtedly an important reason for the persistence of dengue disease [15]. The geographical serotype profile and genotype evolution may contribute to increases in epidemics and disease severity. InDRE has recently made available serotype data by state for 2000–2011 [51]. These data confirm the predominance of DENV-1 and DENV-2 and the increase in the proportion DENV-3 and DENV-4. As noted by Vázquez-Pichardo et al. in their analysis of the 2009–2011 InDRE data [26], outbreaks in new geographic areas and the increased circulation of multiple serotypes in the majority of states is a serious concern. It is well documented that hyperendemicity (the co-circulation of multiple serotypes in a city or country), is believed to be one of the most significant factors influencing dengue severity [52]–[56]. Routine publication of InDRE data could provide an insight into changes in the geographical distribution of dengue virus serotypes and could help understand the patterns and dynamics of virus transmission and the serotypes actively causing disease in Mexico.

### Conclusion

Dengue virus activity in Mexico during the past decade was characterised by more widespread circulation and with a tendency toward reporting increasing numbers of dengue disease cases that began in 2005. All age groups in Mexico are affected by dengue disease, with peak incidence around the age range of 10–20 years and only a gradual decrease with increasing age among adults. High endemicity and the co-circulation of multiple serotypes (as well as increases in laboratory diagnostic support) may contribute to the increased reports of both DF and DHF. These data confirm that dengue disease poses a serious public health problem in Mexico. Despite vector control measures and constant improvements in the diagnosis and management of dengue disease by health services, effective control of the disease has yet to be achieved. Improved epidemiological data from enhanced surveillance strategies (such as incorporating additional sentinel sites, including more private health units, and utilising information technologies) are required to understand the current situation and to enable the evaluation of disease prevention and management interventions. Investigations to provide such data across a broad range of epidemiological settings would include case–control studies of severity of the disease, cohort studies to understand the clinical spectrum of the disease, and studies of seroprevalence and the transmission dynamics of different serotypes.

## Supporting Information

Table S1 Evidence table for sources fulfilling the inclusion and exclusion criteria for the literature review (n = 28).
(PDF)

Table S2 Age distribution of uncomplicated dengue disease in Mexico [20], [30]. Data shown are number of cases (incidence per 100,000 population).
(PDF)

Checklist S1 PRISMA 2009 checklist.
(PDF)
