# Supplementary material for: Epidemiological Trends of Dengue Disease in Mexico (2000–2011): A Systematic Literature Search and Analysis
Source: PLoS Negl Trop Dis. 2014 Nov 6;8(11):e3158. doi: 10.1371/journal.pntd.0003158 (PMC4222737; doi:10.1371/journal.pntd.0003158)
Supplement: Table S1 — Evidence table for sources fulfilling the inclusion and exclusion criteria for the literature review (n = 28). (PDF) [file pntd.0003158.s001.pdf]

Supplementary Table S1: Evidence table for sources fulfilling the inclusion and exclusion criteria for the literature review (n=28)

Citation	Region/geographical area	Study design	Data period: Date range/year	If study:		Summary of data presentation or results/conclusion
				No. patients/ population studied [M:F]	Age range	
San Martín et al., 2010 (10)	Study covered the Americas, but Mexico-specific data were included	Surveillance data: case, incidence and population data obtained from the MoH,	1980–2007		<1–>65	Cases increased over time: from 1,033,417 (1980s) to 2,725,405 (1990s) to 4,759,007 (2000–7). In Mexico between 1980 and 2007 there were 534228 DF cases and 25288 DHF cases; cases peaked between August and October throughout 2000–7 .The male: female ratio in Mexico ranged from 1:1.27 to 1:1.41 (2003–7); children and adolescents (10–14 years of age) had the highest dengue incidence rates followed by adolescents (16–18 years of age).
Diaz et al., 2006 (15)	Mexico	Molecular epidemiological study; retrospective genetic analysis of Dengue viruses isolated in Mexico.	1980–2005	n/a	n/a	The origin of some strains of dengue virus circulating in Mexico could be inferred. The introduction and circulation of different serotypes and genotypes of DENV were associated with changes in the incidence and severity of dengue.
Falcón-Lezama et al., 2009 (16)	Mexico	Surveillance data	Data from 1978–2007 included, focus on 2004–2009			The number of dengue cases from 1978 until 2007 has been characterised by cyclical outbreaks (up to 50 000 cases), followed by years in which the number of cases decreased. Since 1995 all serotypes circulated although the relative frequency of serotypes varied. Increased frequency of DENV-3 isolation between 1996 and 2000 was associated with an increase in morbidity; DENV-2 was most frequent in years with fewer reported cases.
Mexican public health data, 2012–13 (20)	Nationwide data	Surveillance data	2000–2011			Statistical data tables: presentation of Mexican public health data

Citation	Region/geographical area	Study design	Data period: Date range/year	If study:		Summary of data presentation or results/conclusion
				No. patients/ population studied [M:F]	Age range	
Aldana Cruz et al., 2011 (21)	Otomí-Tepehua area	Cross-sectional study (clinical and serological study)	12–18 May 2008	187 cases defined on clinical criteria, found by questionnaire [52:135]	0–>65y	Population movement is a constant risk for the spread of dengue, Spread of cases of dengue began in San Antonio el Grande (n 194), extended to 7 locations, which surround the initial outbreak. The other localities followed the only dirt road leading to San Antonio el Grande.
García Vega, 2009 (22)	Sinaloa: City of Guasave	Clinical study: assessment of the spatio-temporal distribution of *A. aegypti*	April 2008–March 2009			Study mapped the concentration of mosquitoes at various locations throughout the city, and related this to month, temperature and precipitation The highest abundance of females was found in July and August. Two to three months lag between the greatest number of female mosquitoes and the appearance of DF/DHF was established.
International Federation Disaster Relief Emergency Fund, 2009 (23)	Mexico	Surveillance data	2009			News article presenting data from the Epidemiological Surveillance Single Information System (Sistema Único para la Vigilancia Epidemiológica - SINAVE, Mexico). It reports that 41,687 dengue cases were confirmed during 2009, which represents a 30 per cent increase over the previous year. Of these the 7,898 cases were confirmed to be DHF.
Cisneros Solano, 2005 (24)	Oaxaca, Mexico	National surveillance data	1998–2002			Thesis incorporating Dirección General de Epidemiología (DGE) data. Thesis determines under-reporting of cases in Oaxaca using MAC-ELISA, RT-PCR, immunofluorescence and fluorescence-activated cell sorter assays. DENV-2 was found with the highest frequency and greatest distribution, and confirmed to be of the American/Asian genotype.

Citation	Region/geographical area	Study design	Data period: Date range/year	If study: No. patients/ population studied [M:F]	If study: Age range	Summary of data presentation or results/conclusion
Torres et al., 2007 (25)	Latin /Central America, with some Mexico-specific data	Analysis of Latin American surveillance data	Region:1990–2007.Mexico: 2005			Between 1997 and 2007, all countries in the tropical regions of Latin America experienced marked increases in the incidence of both DF and DHF. This broad examination of the health and economic impact of dengue in Latin America reports country-specific data for 2005. Data for Mexico show 16,862 dengue cases in the year, 4,255 cases of DHF and no deaths, with all 4 DENV serotypes circulating during the year.
Vázquez-Pichardo et al., 2011 (25)	Mexico	Serotype identification study	2009–2010	8,349 serum samples analysed (2009: 6,336; 2010: 2,013)	Not specified	2009: 2,944 (46.6%) samples gave positive serotype identification (DENV-1 > DENV-2> DENV-3=DENV-4) 2010:, 1,607 (78.8%) samples gave positive serotype identification. DENV-1 was predominant throughout the country. In 2009, DENV-1 and DENV-2 were identified In 15 states and in10 states only DENV-1was identified. In 2010, DENV-1 and DENV-2 were identified, in 14 states and DENV-1 alone in 9 states.
Brunkard et al., 2008 (27)	Tamaulipas: Matamoros	Autoregressive modelling study to assess influence of climate and weather on dengue incidence (time series analysis)	1995–2005	Not reported	Not specified	Dengue incidence increased by 2.6% one week after every 1ºC increase in weekly maximum temperature and increased 1.9% two weeks after every 1 cm increase in weekly rainfall. Every 1ºC increase in sea surface temperatures was followed by a 19.4% increase in dengue incidence (18 weeks later). Climate and weather play a small but significant role in dengue transmission in Matamoros, Mexico.

Citation	Region/geographical area	Study design	Data period: Date range/year	If study: No. patients/ population studied [M:F]	If study: Age range	Summary of data presentation or results/conclusion
Diarte Arellano, 2007 (28)	State of Sinaloa	Surveillance data	2003–2006			Thesis incorporating Dirección General de Epidemiología (DGE) data. Thesis concludes that dengue is a problem mainly associated with domestic sanitation and that the most important dengue vector, *Ae. aegypti*, can be controlled by physical means and without excessive use of insecticides. Reference is made to a strategy of individual case management and patient selection. An outbreak case-study is provided.
Luzanía Valerio, 2011 (29)	State of Veracruz	Epidemiological spatial analysis study; regional surveillance data	2004–2005			Geographical distribution of DF and DHF cases in the state of Veracruz reported by the state health service.. In 2004, there were 16,148 reported dengue cases throughout the state of Veracruz (14,564 classic dengue and 1,584 DHF). For 2005, 11, 761 cases of dengue were reported (11 128 which were classic dengue and 633 DHF). Regional differences corresponded to the prevailing demographic, ecological and social conditions in the regions.
Navarrete et al., 2002 (30)	Mexico	Surveillance data	1979–2001			This study found variation in annual morbidity rates for dengue from 1979 to 2001. Data relevant to the review are presented in illustrations showing cases of dengue and DHF by epidemiological week (1999-2001),. DHF cases by age group (1999-2001) and DHF mortality according to age groups (1995 to 2001). The authors postulate that co-circulation of the four dengue serotypes may explain the higher occurrence of DF and DHF recorded between 1997 and 2001.

Citation	Region/geographical area	Study design	Data period: Date range/year	If study: No. patients/ population studied [M:F]	If study: Age range	Summary of data presentation or results/conclusion
Rivera Osorio, 2010 (32)	Presumed to be Mexico (whole country)	Clinical study: laboratory serotype data	2006–2007	Not reported	Not specified	This study provided data on the circulation of genotype V of DENV-1 in Mexico. Most isolates were genetically homogeneous, although 13 showed punctual mutations able to be translated as amino acid substitutions in the expressed protein product. No consistent sequences were found in those amino acid differences that correlated with disease severity.
Navarrete-Espinosa et al., 2005 (33)	21 administrative regions or delegations throughout Mexico	Clinical, serological, virological epidemiological study	1995–2003	1,415 cases analysed of 2,743 notified to the Mexican Institute of Social Security; 717 dengue cases reported in 2002. [0.91]	Not specified	Results characterize the clinical profile of DHF cases in Mexico and identify prognostic factors to help clinicians to prevent and properly treat cases of DHF. Cases were divided into two groups: 438 patients with DF, including 109 cases with manifestations of DHF without thrombocytopenia, and 977 cases with DHF, including 79 deaths. 717 dengue cases reported in 2002. The main risk factors associated with mortality were hematemesis and melena.
Loroño-Pino et al., 2004 (34)	Yucatan State	Clinical, serological, virological, nuclear sequencing and phylogenetic study.	Majority of serum samples were collected between 1 Jan 2002 and 31 Dec 31 2002	1,560 patients [53 (54.6%): 44 (45.4%)]; of the 97 confirmed infected patients admitted to hospital)	Not specified	DENV infection was confirmed in 18% (282 of 1,560) of the patients who presented with dengue-like symptoms during this DENV-2 in 2002,. 87 (31%) met the WHO criteria for DHF; (77% of the patients experienced secondary infections in this epidemic. Merida municipality had the highest number of DENV infections (144). Onset of symptoms occurred during August for the majority of confirmed DF and DHF cases.

Citation	Region/geographical area	Study design	Data period: Date range/year	If study:		Summary of data presentation or results/conclusion
				No. patients/ population studied [M:F]	Age range	
Sanchez-Burgos et al., 2008 (36)	Tabasco, Mexico	Transversal seroprevalence study	Sep–Nov 2005	273 students	Not specified	Prevalence of anti-dengue IgG in sera collected from students was 9.1%. Neutralizing antibody frequency: DENV-1: 20%; DENV-2: 100%; DENV-3: 4 %; DENV-4: 68%.The authors observed that DENV-2 circulated more than DENV-3 (even though DENV-3 is more frequently isolated) and an unexpectedly high frequency of neutralizing antibodies against DENV-4 (a rarely isolated serotype in the region).
Espinoza-Gómez et al., 2003 (37)	Colima	Longitudinal clinical, serological, epidemiological study	2001–2002	245 subjects with serological evidence of infection	0–>60y	Over a seven-month period, 12 individuals showed recent dengue infection (incidence: 1.77%); 8 had recent clinical symptoms. There was no evidence of correlation between recent infection and sex, age, or socioeconomic level.
Brunkard et al., 2007 (38)	Brownesville (Texas); Matamoros (Mexico)	Cross sectional serosurvey to assess dengue seroprevalence at the household level	Oct–Nov 2004	300<br>Based on serology: recent infection = 22/300 past infection = 235/300	15–>75y	Recent dengue infection was detected in residents of Brownsville (2%) and Matamoros (7.3%). Past infection was detected in of Brownsville residents (40%) and Matamoros residents (78%). Presence of larval habitat, absence of air-conditioning and street drainage, and weekly family income ≤$100, were risk factors that predicted past dengue infection.
Ramos et al., 2008 (39)	Tamaulipas: Metamoros	Cross-sectional household survey of serological, entomological and epidemiological risk factors	5–10 Dec 2005	132 participants in 111 households [45:85]	5 –65+y	The estimated incidence of recent dengue infection was 32%; the estimated prevalence of past dengue infection was77%. The Breteau index of 28 reflects an abundant winter population of *Aedes* mosquitoes. The largest categories of infested containers were waste tyres and buckets..

Citation	Region/geographical area	Study design	Data period: Date range/year	If study: No. patients/ population studied [M:F]	If study: Age range	Summary of data presentation or results/conclusion
Navarrete-Espinosa et al., 2006 (40)	Veracruz: Jaltipan	Clinical, epidemiological, cross-sectional study to determine prevalence of previous dengue using anti-dengue IgG serology	2003	Population studied: 500	1–65y	The seroprevalence for dengue virus in the general population was 79.6% and slightly higher in women (81%) than men (78%).Seroprevalence was 85%, in those with a previous dengue infection compared with 79%; in those without previous infection. A direct relationship was observed with age: individuals aged 15–24 years had an almost three times higher prevalence than children under 5 years of age.
Cisneros-Solano et al., 2004 (41)	Oaxaca (10 sites)	Clinical, serological and virological cross-sectional epidemiological study	July 2000–Nov 2001	100 patients with clinical signs of dengue	All ages	94/100 serum samples suspected of dengue were confirmed positive; The number of females infected was higher than the number of males, and the most affected age group was people aged under 35 years. The study also highlighted that the sensitivity and specificity of diagnostic tools were crucial for epidemiological studies.
Günther, 2007 (42)	State of Oaxaca	Clinical study: epidemiological analysis, plus serotype data using serum from dengue patients	2003–2006	Study of confirmed cases	Not specified	Increase in the number of dengue cases observed, from 190 in 2004 to 3400 in 2006. Those aged 11–15 years were most affected (968 cases between 2003 and 2006). A greater number of women were diagnosed with DF than men, although more men were diagnosed with DHF than women (14.9% vs 11.7%). All four serotypes were detected. Of 109 sera examined, 65 were positive, and 44 negative. DENV-4 was the most frequently occurring.
Günther et al., 2009 (43)	Oaxaca state: all six sanitary jurisdictions of Oaxaca	Clinical, serological, virological study (109 patients from 2005 also had molecular virology studies)	2004– 2006	4554 patients clinical symptoms of dengue [2,170:2,384]	All	Substantial increase in the number of dengue cases observed during the study period. The most affected region was coastal, where the climatic conditions were ideal for vector development and where there is significant migratory activity. The most affected age group was aged 11–15 years. In the 16–50 your age group, DHF was more frequent in men (19.6%) than in women (10.8%) ($p<0.001$). All four serotypes were detected.

Citation	Region/geographical area	Study design	Data period: Date range/year	If study:		Summary of data presentation or results/conclusion
				No. patients/ population studied [M:F]	Age range	
Ramírez-Zepeda et al., 2009 (44)	Sinaloa: Culiacan	Cross-sectional, observational clinical and epidemiological study	1 Oct–2 Dec 2003	241 patients met the study criteria:207 DF; 34 DHF [101:140]	<25–>65y (mean: 34.7 ± 15.1 year).	Documents the clinical and epidemiological characteristics of patients with dengue treated at a hospital in Culiacan, Sinaloa.. Multivariate analysis, adjusted for age, sex and the presence of dengue cases locally, suggested that the variables predictive of increased disease severity were: (a) the presence of ascites, hematemesis, thrombocytopenia, conjunctival hyperaemia, persistent vomiting and (b) the absence of nasal congestion.
Espinoza-Gomez et al., 2005 (45)	Colima	Case series: clinical, serological, virological	2002	1,766 DF, 617 DHF identified by the Health Secretary of Colima [152:137]; of the confirmed hospitalised cases	0–75y	Of the 1,079 patients hospitalized, dengue was confirmed by laboratory testing in 289. The majority presented clinical characteristics consistent with DHF; six died of consequences attributed to dengue (0.8%). DENV-2 was isolated in 12 cases.
Anguiano Moreno et al., 2011 (46)	Colima	Surveillance data	2008–2010			Colima is the major contributor to the total number of dengue cases in Mexico, and the western region contributed 45% to the 2009 total. Strategies to combat dengue in Colima were effective: 81.4% reduction in the number of dengue cases over the previous year.
