# Supplementary material for: Epidemiological Trends of Dengue Disease in Mexico (2000–2011): A Systematic Literature Search and Analysis
Source: PLoS Negl Trop Dis. 2014 Nov 6;8(11):e3158. doi: 10.1371/journal.pntd.0003158 (PMC4222737; doi:10.1371/journal.pntd.0003158)
Supplement: Table S2 — Age distribution of uncomplicated dengue disease in Mexico [20], [30]. Data shown are number of cases (incidence per 100,000 population). (PDF) [file pntd.0003158.s002.pdf]

Supplementary Table S2. Age distribution of uncomplicated dengue disease in Mexico.[19,29] Data shown are number cases (incidence per 100,000 population).

Year	Age group (years)										
	**<1**	**1–4**	**5–9**	**10–14**	**15–19**	**20–24**	**25–44**	**45–49**	**50–59**	**60–64**	**≥65**
2000	9 (0·42)	54 (0·62)	206 (1·84)	233 (2·10)	213 (2·03)	144 (1·48)	580 (1·99)	81 (1·90)	112 (1·87)	27 (1·26)	54 (1·13)
2001*	(4·8)				(5·7)		(5·1)	(4·4)			(1·5)
2001	34 (1·62)	257 (3·01)	553 (4·98)	635 (5·72)	495 (4·66)	424 (4·34)	1460 (4·89)	216 (4·84)	325 (5·20)	92 (4·14)	147 (2·97)
2002	69 (3·34)	516 (6·12)	1664 (15·12)	2260 (20·35)	1732 (16·16)	1184 (12·05)	3746 (12·24)	638 (13·68)	754 (11·59)	250 (10·90)	355 (6·90)
2003	27 (1·36)	90 (1·09)	334 (2·98)	726 (6·43)	655 (6·11)	500 (4·98)	1743 (5·60)	311 (6·17)	415 (5·99)	134 (5·59)	207 (3·96)
2004	36 (1·84)	179 (2·22)	457 (4·14)	980 (8·64)	725 (6·74)	550 (5·44)	1991 (6·28)	412 (7·84)	486 (6·73)	152 (6·14)	193 (3·56)
2005	31 (1·60)	402 (5·08)	1499 (13·90)	3042 (26·80)	2326 (21·51)	1500 (14·75)	5393 (16·70)	979 (17·92)	1306 (17·30)	378 (14·75)	573 (10·18)
2006	82 (4·29)	650 (8·34)	2419 (22·96)	4629 (40·90)	4053 (37·27)	2436 (23·84)	5943 (18·10)	1485 (26·21)	1415 (17·93)	559 (21·08)	715 (12·25)
2007	155 (7·98)	1289 (16·27)	4483 (40·81)	8338 (76·58)	6739 (64·35)	3897 (40·46)	10,910 (34·23)	1909 (33·67)	2643 (33·31)	806 (29·92)	1435 (24·82)

Year	Age group (years)										
	<1	1–4	5–9	10–14	15–19	20–24	25–44	45–49	50–59	60–64	≥65
2008	71 (3·69)	770 (9·91)	2550 (23·62)	4879 (45·03)	4638 (44·24)	2710 (27·97)	6862 (21·27)	1200 (20·44)	1876 (22·66)	1091 (39·06)	1265 (21·14)
2009	898 (46·97)	2220 (28·96)	8505 (80·87)	18 056 (167·01)	19 774 (188·75)	13 482 (138·43)	36,755 (112·63)	6229 (102·70)	8405 (97·30)	2256 (77·82)	4069 (65·69)
2010	367 (19·33)	962 (12·67)	3047 (29·97)	5674 (52·39)	6114 (58·49)	4338 (44·32)	10,051 (30·48)	1704 (27·24)	2423 (26·89)	692 (22·98)	1368 (21·33)
2011	218	443	1167	2161	2573	1800	4470	691	1065	321	515
	(11·6)	(5·9)	(11·9)	(19·9)	(24·7)	(18·3)	(13·4)	(10·7)	(11·3)	(10·3)	(7·8)

Mexican public health data [19] except *Navarette, 2002 [29]
